# Comparison between two tomographic sections in the diagnosis of external root resorption

**DOI:** 10.1590/S1678-77572010000300019

**Published:** 2010

**Authors:** Cláudio Afonso LERMEN, Gabriela Salatino LIEDKE, Heloísa Emília Dias da SILVEIRA, Heraldo Luis Dias da SILVEIRA, Alessandro André MAZZSOLA, José Antônio Poli de FIGUEIREDO

**Affiliations:** 1 BDS, MSc Postgraduate student, Department of Surgery and Orthopedics, School of Dentistry, Federal University of Rio Grande do Sul, Porto Alegre, RS, Brazil.; 2 BDS, Postgraduate student, Department of Surgery and Orthopedics, School of Dentistry, Federal University of Rio Grande do Sul, Porto Alegre, RS, Brazil.; 3 BDS, PhD, Associate Senior Lecturer, Department of Surgery and Orthopedics, School of Dentistry, Federal of University of Rio Grande do Sul, Porto Alegre, RS, Brazil.; 4 BDS, PhD, Lecturer, Department of Surgery and Orthopedics, School of Dentistry, Federal of University of Rio Grande do Sul, Porto Alegre, RS, Brazil.; 5 MSc, Physicist and Coordinator of the Imaging Center of ‘Moinhos de Vento’ Hospital, Porto Alegre, RS, Brazil.; 6 BDS, MSc, PhD, Senior Lecturer of Endodontology and Dean of the Postgraduate Program in Dentistry, Pontifical Catholic University of Rio Grande do Sul, Porto Alegre, RS, Brazil.

**Keywords:** Tomography, External root resorption, Diagnosis

## Abstract

**Objectives:**

To assess the accuracy of coronal and sagittal CT sections to detect cavities simulating root resorption.

**Material and Methods:**

60 mandibular incisors were embedded in plaster bases, and cavities with 0.6, 1.2 or 1.8 mm in diameter and 0.3, 0.6 or 0.9 mm in depth (small, medium and large cavities) were drilled on the buccal surfaces with high-speed round burs with diameters of 0.6, 1.2 and 1.8 mm to simulate external inflammatory root resorption. Simulations in the cervical, middle and apical thirds of each tooth root were made randomly. The Dental Scan software was used to obtain 1-mm-thick axial images from direct scanning, which were reconstructed in the coronal and sagittal planes using 3D software (Syngo FastView). Each series was loaded into the software. Fourteen images of each tooth were reconstructed in the coronal plane and 14 in the sagittal plane. A total of 1,652 images were obtained for analysis. Series information, tooth number and the plane reconstructed were stored. The images generated were saved on a CD-ROM together with the visualization software (Syngo FastView). Images were analyzed by a previously calibrated blinded, radiologist. Cochran’s Q test was conducted separately for each region analyzed followed by pair-wise comparison by the McNemar test (p=0.05).

**Results:**

No statistically significant difference (p>0.05) was observed in the diagnosis of simulated resorption between the apical, middle, and coronal thirds. When the axial plane was assessed separately, diagnoses were statistically different (p<0.05) among the three root thirds. The apical third differed significantly (p<0.05) from the cervical and middle thirds. Diagnostic errors were more often observed in the apical third compared to the cervical and middle thirds. Mid-sized cavities revealed no statistically significant differences (p>0.05) between planes, irrespective of the third in which the resorptions were located.

**Conclusion:**

When tomographic sections are requested for the diagnosis of buccal or lingual external root resorption, sagittal sections afford the best image characterization of the resorption process.

## INTRODUCTION

Radiographs obtained at different angles may be useful to determine which surface is affected by external root resorption, but they do not provide a representation of the three dimensions of real resorption defects^2,7^. Their actual location also interferes in the radiographic visualization: resorption areas on the buccal or lingual surfaces are more difficult, if not impossible, to visualize. Yet, this is the opposite of what radiographic inspection affords regarding the mesial and distal surfaces^13^.

Computed tomography (CT) has been used to obtain a better visualization of invasive root resorption, and is a useful resource to provide an accurate diagnosis of the extension and location of the resorption^8^. Although it is not always necessary, CT can be very helpful in some cases to overcome the difficulties of conventional radiography is distinguishing the lesion from normal anatomic structures. This is advantageous to both patients and clinicians for addressing the treatment strategy more comprehensively.

CT affords obtaining images without overlapping of structures. In a previous study investigating CT diagnostic ability to evaluate simulated resorption lesions^13^ , it was difficult to identify small cavities in the apical third. We hypothesize that sagittal planes could enhance the visualization ability of CT. The present study aimed to assess the accuracy of coronal and sagittal CT sections to detect cavities simulating root resorption.

## MATERIAL AND METHODS

Sixty human mandibular incisors were selected from the Tooth Bank of the Department of endodontics of the School of Dentistry of the Federal University of Rio Grande do Sul, Brazil after approval by the local Research ethics Committee and randomly numbered from 1 to 60. The teeth were not sectioned, but their root segments were divided into thirds -cervical, middle and apical -totalizing 180 root thirds. For each third (cervical, middle and apical) there were 4 simulation possibilities: small, medium or large cavity, or no cavity. Thus, there were 12 possible "third x cavity size" combinations, and 15 teeth were randomly assigned to each combination.

To simulate external root resorption, the teeth were placed in plaster bases, and cavities of 0.6, 1.2 or 1.8 mm in diameter and 0.3, 0.6 or 0.9 mm in depth (small, medium and large cavities) were drilled according to a protocol reported in the literature^1,4,5,6^. The cavities were drilled on the buccal surfaces with high-speed round burs with diameters of 0.6, 1.2 and 1.8 mm (KG, Sorensen, São Paulo, SP, Brazil), which were adapted to a device that ensured standardization of cavity diameter and depth. Simulations in the cervical, middle and apical thirds of each root were made randomly.

One tooth fractured and was discarded. The 59 remaining teeth, divided into 2 groups of 20 specimens each and one group of 19 specimens, were fixed in 1-cm-thick wax plates and placed over a cylindrical plastic container with water. This assembly was then placed on the Single-Slice TS (Somaton emotion Duo, Siemens, erlangen, Germany) scan table. The use of wax and water was tested in a pilot-study to simulate tissues with water and fat density, and thus avoid differences in density between air and teeth and reduce artifacts in the image. The Dental Scan software was used to obtain 1-mm-thick axial images, obtained from direct scanning, at 1.5 x 0.5 mm reconstruction intervals along the whole tooth extension, according to the basic software protocol 120 kVp, 80 mA, 1 s rotation time. The axial sections of the sets of teeth were reconstructed in the coronal and sagittal planes using the 3D software (Syngo FastView, Siemens Medical, Germany). each series was loaded into the software. The volume of interest definition tool (VOI Clip Box) was used to isolate each tooth, so as to allow positioning of sagittal and coronal sections. Reconstructions were made at section thickness of 1.0 mm and section intervals of 0.5 mm. Roughly, 14 images of each tooth were reconstructed in the coronal plane and 14 in the sagittal plane. A total of 1,652 images were obtained for analysis. Series information, tooth number and the plane reconstructed were stored. The images generated were saved on CD-ROM together with the visualization software (Syngo FastView).

Images were analyzed by a previously calibrated, blinded radiologist. In order to verify statistically significant differences among the results for the cervical, middle, and apical thirds in the resorption diagnosis, the Cochran's Q test was used followed by the McNemar test for pair-wise comparisons. In the same way, the three cavity sizes were analyzed independently for the cervical, middle and apical thirds, and for the three planes (axial, coronal and sagittal). In all cases, a 5% significance level was adopted to demonstrate a likely enhancement in the diagnostic capacity of coronal and sagittal sections by the 3D software, as compared to axial sections.

## RESULTS

The total number of simulated resorptions, number of simulated resorptions diagnosed by CT, and the respective percent values in coronal and sagittal sections are shown in Table 1, similarly to the representation for axial sections^5^.

**Table 1:** Total number of simulated resorptions, number of simulated resorptions diagnosed by CT, and the respective percent values in coronal and sagittal sections

**Root third**	**Simulated Resorption**		**Diagnosed resorption**		**%**	
	**Coronal**	**Sagittal**	**Coronal**	**Sagittal**	**Coronal**	**Sagittal**
						
Apical	44	44	38	42	86.36	95.45
Middle	44	44	41	43	93.18	97.27
Cervical	43	43	41	42	95.34	97.67
Total	131	131	120	127	91.6	96.94

Simulated and diagnosed resorptions for coronal and sagittal sections (Table 2) were expressed in more detail, considering not only the location (apical, middle and cervical thirds), but also the size of simulated resorptions (small, midium, and large) for each third individually. Small resorptions in the apical third were diagnosed in 57.14% of the cases in the coronal sections, and in 85.71% of the cases in the sagittal sections.

**Table 2:** Comparison between simulated and diagnosed resorptions in the apical, middle and cervical thirds, and respective resorption sizes, in coronal and sagittal sections

**Rooth Third**	**Size**	**Simulated resorptions**		**Diagnosed resorptions**		**%**	
		**Coronal**	**Sagittal**	**Coronal**	**Sagittal**	**Coronal**	**Sagittal**
							
	Small	14	14	8	8	57.14	86.71
Apical	Medium	15	15	15	15	100	100
	Large	15	15	15	15	100	100
	Total	44	44	42	42	86.36	95.45
	Small	14	14	14	14	85.71	100
Middle	Medium	15	15	14	14	93.33	93.33
	Large	15	15	15	15	100	100
	Total	44	44	43	43	93.18	97.72
	Small	14	14	13	13	92.85	92.85
Cervical	Medium	15	15	14	14	93.33	93.33
	Large	14	14	14	14	100	100
	Total	43	43	41	41	95.34	95.34

**Figure 1:**
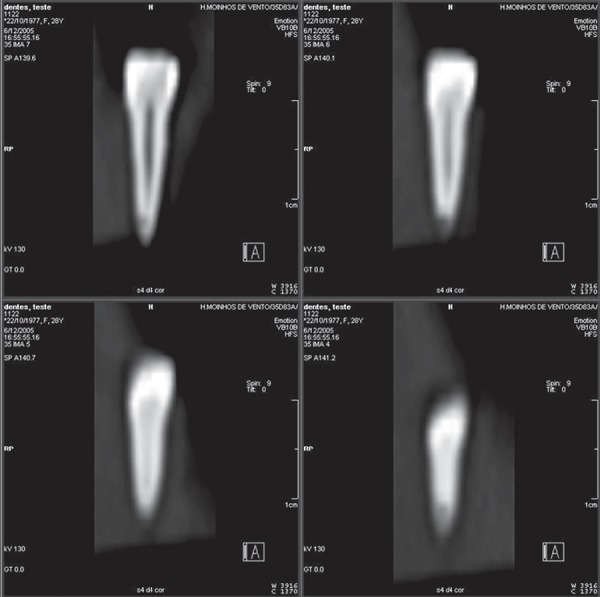
Series of coronal CT sections showing cavities on apical and middle thirds

**Figure 2:**
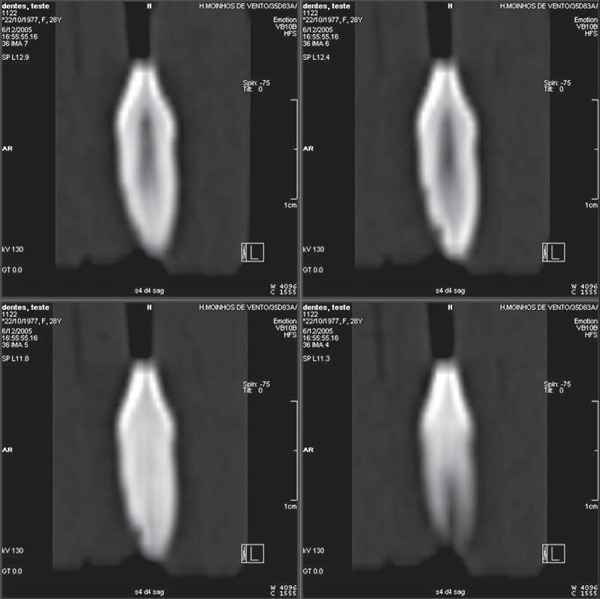
Series of sagittal CT sections showing cavities on apical and middle thirds

In the coronal sections (Figure 1), 120 of the 131 simulated resorptions were successfully observed, which represents a total of 91.60% correct diagnoses. The lowest percentage of correct diagnoses (86.36%) was observed in the apical third (Table 1). Similarly, in the sagittal sections (Figure 2), 127 of the 131 simulated resorptions were identified, which represents a total of 96.94% correct diagnoses. Again, the lowest percentage of correct diagnoses (95.45%) was observed in the apical third (Table 1).

The increase in cavity size led to an enhanced diagnostic capacity of CT. In general, the cavities in the apical third were the most difficult to diagnose.

Based on the results of the Cochran’s Q test for coronal and sagittal planes, no statistically significant difference (p>0.05) was observed in the diagnosis of simulated resorption cavities among the apical, middle, and coronal thirds. Yet, when the axial plane was assessed separately, diagnoses were statistically different (p<0.05) among the three thirds of the root. Based on the results of the McNemar test, the apical third differed significantly (p<0.05) from the cervical and middle thirds. Diagnostic errors were more often observed (p<0.05) in the apical third, compared to the cervical and middle thirds.

The Cochran’s Q test for the mid-sized cavities revealed no statistically significant differences (p>0.05) among the planes, irrespective of the third in which resorption was located. As no diagnostic errors were observed for large cavities, the Cochran’s Q test was not necessary. No statistically significant difference (p>0.05) was observed for small cavities between the cervical and middle thirds. Nevertheless, the apical third showed a significant difference (p<0.05) between axial and sagittal planes, with the former having a higher occurrence of misdiagnoses.

## DISCUSSION

Conventional and digital radiographs have limitations for the accurate diagnosis of external root resorptions, specially when manifested as small defects located on the buccal or lingual surfaces^1,3,5,9,13,15^. Silveira, et al.^13^ (2003) used a dental CT with images of axial sections and revealed that it enhanced the capacity to diagnose simulated tooth resorption, as compared to conventional radiographs. The high specificity of CT (100%), though, was proved by the absence of false-positive results. Conversely, conventional radiograph has been shown to produce false-negative results in 15.3% of the cases examined in another study investigating the diagnosis of external root resorption using tuned-aperture computed tomography^9^. However, major difficulties are found when using axial sections to detect small cavities located on the apical root third^13^.

By using the other planes (coronal and sagittal) for diagnostic imaging and then comparing the results with previous data^13^ , the present study observed that the findings are quite similar in terms of diagnostic capacity of mid-sized and large cavities simulating resorptions. The percentage of closely comparable results was high, independently of the root third and the plane examined. Nevertheless, the largest differences are observed as far as small simulated resorptions in the apical plane are considered. Silveira, et al.^13^ found that only 28.57% of these resorptions were seen in axial sections, while 57.14% were observed in coronal sections, and 85.71% in sagittal sections, with significant differences between the axial and sagittal planes.

The present results also showed a rapid increase in the characterization of resorptions for the apical third, which is a region that typically imposes the greatest difficulties to the diagnosis of resorptions due to the peculiar features as narrowing of the root and reduced area. The axial plane was the least precise in affording resorption identification, followed by the coronal and the sagittal planes. As demonstrate elsewhere^11^ , clinicians must know the best protocol to resort for an accurate diagnosis when requesting CT scans.

The increased diagnostic capacity in the sagittal plane may be explained by the broader and more specific aspects afforded. The sagittal plane reveals several important landmarks and features of the dental structures, such as the cementoenamel junction and root morphology, which helps locating cavities across the root thirds, especially small ones, and facilitates the diagnostic process.

The introduction of cone beam computed tomography (CBCT) for maxillofacial region provided the same characteristics of interaction with data already observed in CT^14^ , but with lower radiation doses, as compared to MSCT^12^. This new technique has proved its value for endodontic diagnosis^9^. Thus, in agreement with other authors^7^ , CT technique should be adopted with care and when strictly needed, that is, in cases of small resorption cavities, especially those located on the buccal or lingual root surfaces, which conventional radiographs are not suitably efficient to detect and if a CBCT device is not available. Additionally, exposure to radiation may be minimized by reducing milliamperage and rotation time, and by using a pitch factor 2^11^.

## CONCLUSION

When tomographic sections are requested for the diagnosis of buccal or lingual external root resorption, sagittal sections afford the best image characterization of the resorption process.
